# Isomer Profiles of Perfluorochemicals in Matched Maternal, Cord, and House Dust Samples: Manufacturing Sources and Transplacental Transfer

**DOI:** 10.1289/ehp.1003265

**Published:** 2011-07-14

**Authors:** Sanjay Beesoon, Glenys M. Webster, Mahiba Shoeib, Tom Harner, Jonathan P. Benskin, Jonathan W. Martin

**Affiliations:** 1 Division of Analytical and Environmental Toxicology, Department of Laboratory Medicine and Pathology, University of Alberta, Edmonton, Alberta, Canada; 2 Centre for Health and Environment Research, School of Environmental Health, University of British Columbia, Vancouver, Canada; 3 Science and Technology Branch, Environment Canada, Toronto, Ontario, Canada

**Keywords:** isomers, perfluorochemicals, PFOA, PFOS, transplacental transfer

## Abstract

Background: Perfluorochemicals (PFCs) are detectable in the general population and in the human environment, including house dust. Sources are not well characterized, but isomer patterns should enable differentiation of historical and contemporary manufacturing sources. Isomer-specific maternal–fetal transfer of PFCs has not been examined despite known developmental toxicity of perfluorooctane sulfonate (PFOS) and perfluorooctanoate (PFOA) in rodents.

Objectives: We elucidated relative contributions of electrochemical (phased out in 2001) and telomer (contemporary) PFCs in dust and measured how transplacental transfer efficiency (TTE; based on a comparison of maternal and cord sera concentrations) is affected by perfluorinated chain length and isomer branching pattern.

Methods: We analyzed matching samples of house dust (*n* = 18), maternal sera (*n* = 20), and umbilical cord sera (*n* = 20) by isomer-specific high-performance liquid chromatography tandem mass spectrometry.

Results: PFOA isomer signatures revealed that telomer sources accounted for 0–95% of total PFOA in house dust (median, 31%). This may partly explain why serum PFOA concentrations are not declining in some countries despite the phase-out of electrochemical PFOA. TTE data indicate that total branched isomers crossed the placenta more efficiently than did linear isomers for both PFOS (*p* < 0.01) and PFOA (*p* = 0.02) and that placental transfer of branched isomers of PFOS increased as the branching point moved closer to the sulfonate (SO_3_^–^) end of the molecule.

Conclusions: Results suggest that humans are exposed to telomer PFOA, but larger studies that also account for dietary sources should be conducted. The exposure profile of PFOS and PFOA isomers can differ between the mother and fetus—an important consideration for perinatal epidemiology studies of PFCs.

The most prominent perfluorochemicals (PFCs) in human samples are perfluorooctane sulfonate (PFOS), perfluorooctanoate (PFOA), and perfluorohexane sulfonate (PFHxS), yet the sources and pathways of human exposure to these, and other PFCs are not well characterized. Perfluorinated acids are ubiquitous in the global environment, owing to their long history of manufacture and resistance to biological and environmental degradation pathways. Specifically for PFOA, the manufacturing sources responsible for its presence in various environments are not well understood, and future human exposure is therefore difficult to predict. There are two main manufacturing methods leading to PFOS and PFOA: electrochemical fluorination (ECF) and telomerization. The 3M Company manufactured the bulk of PFOS (and higher-molecular-weight precursor materials), PFHxS, and PFOA by ECF until 2001, at which time they voluntarily phased out these chemistries. Nonetheless, PFOS and its precursors continue to be manufactured by other companies in Asia (Martin et al. 2010). Telomerization continues to be used to manufacture PFOA. ECF and telomerized PFOA can be readily distinguished analytically because ECF PFOA consists of a mix of linear and branched isomers (Loveless et al. 2006; Reagen WKL, Jacoby CB, Purcell RG, Kestner TA, Payfer RM, et al., unpublished data), whereas telomerized PFOA is almost exclusively the linear isomer (Kissa 1994).

If humans are exposed predominantly to ECF sources of PFOS and PFOA, serum concentrations should be decreasing because of their phase-out. In fact, when the 3M Company stopped manufacturing PFOS and PFOA by its ECF technique, blood levels of PFOS declined steadily in Americans. However, for PFOA the initial rate of decline was much less than anticipated (Olsen et al. 2008), and the most recent data from the National Center of Health Statistics of the U.S. Centers for Disease Control and Prevention (Kato et al. 2011) indicate that serum PFOA did not decline between April 2003 and August 2007 and may be increasing [see Supplemental Material, Figure 1 (http://dx.doi.org/10.1289/ehp.1003265)]. This suggests that exposure to recently produced telomer sources of PFOA might be important, but the relative importance of ECF- and telomer-derived PFC exposures through different exposure pathways (e.g., diet, dust, water, air) is unknown. Nonetheless, the potential for telomer PFOA exposure is recognized, and in 2006 a global stewardship program was implemented to reduce emissions of this chemical [U.S. Environmental Protection Agency (EPA) 2006].

**Figure 1:**
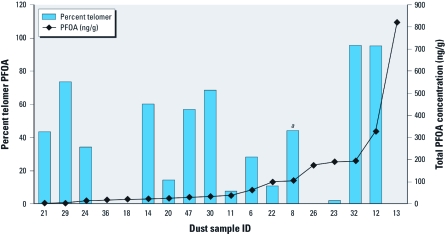
Percent telomer PFOA and total PFOA concentration in house dust samples (left to right, lowest to highest total PFOA concentration), by ID (identification number). ***^a^***Sample collected by mechanical sweeper instead of vacuum.

For many environmental chemicals, house dust can be a major source of exposure (Butte and Heinzow 2002), particularly for children (U.S. EPA 2008). For PFOS and PFOA, food is a major source of exposure, but house dust can also be important under scenarios of high dust ingestion (Bjorklund et al. 2009; Goosey and Harrad 2011; Haug et al. 2011; Shoeib et al. 2011; Tittlemier et al. 2007). Thus, it is important from a risk mitigation perspective to understand whether PFCs in house dust are from current or historical manufacturing sources. PFOS and PFOA have been measured in dust previously (Kato et al. 2009; Kubwabo et al. 2005), but isomer-specific PFC analytical methods (Benskin et al. 2007; Langlois and Oehme 2006) have not been used to determine the manufacturing origins of PFOA and other PFCs in house dust.

Loveless et al. (2006) demonstrated that linear ammonium PFOA was generally more toxic than branched PFOA, but the isomer-specific toxicity of PFCs has not been examined because of the lack of available standards. Studies in rats and zebrafish show that PFOA and PFOS are developmental toxicants (Lau et al. 2004), and many human epidemiology studies are now emerging on the potential perinatal effects of PFCs. For example, some epidemiology studies have shown inverse associations between PFOA exposure and birth weight (Apelberg et al. 2007; Fei et al. 2008), whereas others did not find an association (Monroy et al. 2008; Nolan et al. 2009). Furthermore, other adverse human health effects associated with PFCs are being detected in both background (Nelson et al. 2010) and highly exposed populations (Steenland et al. 2010). From a public health perspective, and recognizing that many PFCs occur as multiple isomers of unknown relative toxicity, it may be important to characterize the exact nature of PFC exposure to humans, including for the mother and the fetus.

Understanding the maternal–fetal transmission of PFCs is necessary to clearly understand the risks and mechanisms of human developmental toxicity. Of studies that have reported the maternal–fetal transfer of PFCs, Hanssen et al. (2010) produced the only study to examine branched isomers separately from linear isomers. However, individual branched isomers were not examined separately (i.e., total branched PFOS was compared with linear PFOS). Although we are beginning to understand the pharmacokinetic properties of specific branched PFCs in animal models (De Silva et al. 2009), no study has yet investigated isomer-specific PFC pharmacokinetics in humans. In an attempt to understand the transplacental transfer of PFCs (mainly PFOS and PFOA), multiple studies have tested maternal and umbilical cord blood samples from different populations (Fei et al. 2007; Fromme et al. 2010; Hanssen et al. 2010; Inoue et al. 2004; Kim et al. 2011; Midasch et al. 2007; Monroy et al. 2008; Needham et al. 2011). One consistent finding in all these studies is that cord serum has lower total PFOA and lower total PFOS than does maternal serum; however the isomer-specific transplacental transfer of the various branched isomers has not been examined despite evidence that the placental transfer is greater for total branched PFOS isomers than for linear PFOS (Hanssen et al. 2010).

In the present study we collected dust from the homes of 20 pregnant women who also donated a blood sample at 15 weeks of gestation and a cord blood sample at delivery. We measured PFC concentrations and isomer profiles in all samples in an effort to identify sources of PFCs in house dust and to examine the isomer-specific transfer of PFCs across the placenta.

## Materials and Methods

*Nomenclature and acronyms.* For structural isomers, we use the nomenclature defined by Benskin et al. (2007). Using PFOS as an example, the following annotations are used to represent the structure of each isomer based on relative position of perfluoromethyl substitution: linear perfluorooctane sulfonate (*n*-PFOS), perfluoroisopropyl (*iso*-PFOS), 5-perfluoromethyl (5*m*-PFOS), 4-perfluoromethyl (4*m*-PFOS), 3-perfluoromethyl (3*m*-PFOS), 1-perfluoromethyl (1*m*-PFOS), *tert*-perfluorobutyl (*tb*-PFOS), and sum of all dimethyl isomers (Σ*m*_2_-PFOS). Except for *n*-PFOS, all of the above-mentioned isomers are branched isomers [for structures, see Supplemental Material, Figure 2 (http://dx.doi.org/10.1289/ehp.1003265)].

**Figure 2:**
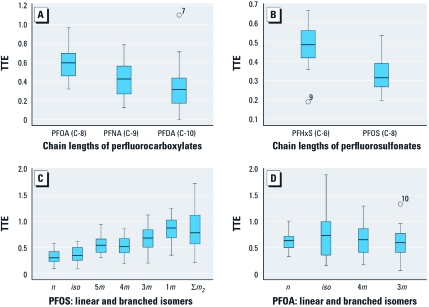
TTE distributions for different-chain-length perfluorocarboxylates (*A*) and perfluorosulfonates (*B*) and for linear and branched PFOS (*C*) and PFOA (*D*) isomers. The upper and lower bounds of the boxes indicate the 75th and 25th percentiles, respectively, and the horizontal lines within the boxes indicate median values. The upper and lower limits of the whiskers indicate minimum and maximum values, respectively, and points above or below the whiskers indicate outlier values. In *A*, *B*, and *D*, the number attached to each outlier is the number of a specific sample.

*PFC chemical standards.* The 3M Company donated ECF PFOS [30% branched and 70% linear, by ^19^F nuclear magnetic resonance (NMR)] and PFOA (22% branched and 78% linear, by ^19^F NMR) standards (Reagen WKL, Jacoby CB, Purcell RG, Kestner TA, Payfer RM, et al., unpublished data). All other PFC standards, including PFOS and PFOA isomer standards and linear mass-labeled internal standards for perfluorobutanoate, perfluorohexanoate (PFHxA), PFHxS, PFOA, perfluorononanoate (PFNA), PFOS, perfluorodecanoate (PFDA), perfluoroundecanoate (PFUnA), and perfluorododecanoate (PFDoA), were obtained from Wellington Laboratories (Guelph, ON, Canada).

*Blood collection.* Samples (*n* = 20) analyzed in this study are a subset of the Chemicals, Health and Pregnancy (CHirP) cohort recruited in 2007–2008 in Vancouver (BC, Canada) (Webster et al. 2011). All participants provided informed consent. Laboratory personnel collected blood samples from pregnant volunteers in Vancouver at 15 weeks of gestation, and samples of cord blood (*n* = 20) were collected at delivery. After serum separation, all samples were stored at –80°C. Ethical clearance was obtained from the research ethics boards of the University of British Columbia, the University of Alberta, Health Canada, and the three participating hospitals.

*Serum preparation.* The method of Kuklenyik et al. (2004) was adapted for the extraction of PFCs from serum using a Rapid Trace system (Caliper Life Sciences, Hopkinton, MA, USA). The solid-phase extraction cartridge (Oasis-HLB, Waters, Milford, MA, USA; 60 mg/3 mL) was conditioned with 2 mL methanol followed by 2 mL of 0.1 M formic acid. Serum was prepared for extraction by mixing 3 mL of 0.1 M formic acid with 1.0 mL serum. After the addition of mass-labeled internal standards (10 ng each), the mixture was vortexed and sonicated for 20 min. Prepared serum was added to the column and washed successively with 3 mL of 0.1 M formic acid, 6 mL of 50% 0.1 M formic acid/50% methanol, and 1 mL of 1% ammonium hydroxide. The cartridge was drained by vacuum, and PFCs were eluted with 1.0 mL of 1% ammonium hydroxide in acetonitrile. The eluate was concentrated to 100 μL followed by the addition of 200 μL of 90% 20 mM acetic acid in 10% methanol. Method blanks containing calf serum, a calf serum sample spiked at 0.5 ng/mL of each PFC, and a human serum sample spiked at 10 ng/mL were analyzed with the study samples.

*Dust collection and extraction.* Dust samples were also a subset of those collected for the larger CHirP study (Shoeib et al. 2011). At 20–24 weeks of gestation, participants donated a used vacuum cleaner bag from their vacuum cleaner, or we took grab samples from the participants’ bagless vacuum cleaners (*n* = 18). Samples were stored at –20°C, and before analysis a portion of each dust sample was sieved using a stainless-steel sieve (mesh size, 150 μm; VWR International, Montreal, QC, Canada). Sieved dust (0.1 g) was spiked with 3.3 ng mass-labeled internal standards; 4 mL methanol was added, and the sample was vortexed for 5 min, sonicated for 1 hr, and centrifuged at 3,400 rpm for 10 min. A 2-mL aliquot was reduced by evaporation to 100 μL, and 200 μL of 20 mM acetic acid with 10% methanol was added before high-performance liquid chromatography tandem mass spectrometry (HPLC-MS/MS).

*Total PFC analysis.* For total PFC concentrations, separation was by HPLC on a 150-mm Synergi Hydro-RP C-18 column (Phenomenex, Torrance, CA, USA). Gradient elution at 600 μL/min used A [20 mM ammonium acetate (pH 4) in water] and B (methanol) mobile phases. Initial conditions were 60% A for 1 min, ramped to 20% A by 3 min, a 5-min hold, an increase to 100% B by 8.5 min, and a hold until 14 min, at which time initial conditions were reestablished. MS/MS data were collected on an Applied Biosystems API 3000 mass spectrometer (Applied Biosystems, Carlsbad, CA, USA) using electrospray ionization in negative-ion mode [for mean recoveries of total PFCs in serum and dust, see Supplemental Material, Table 1 (http://dx.doi.org/10.1289/ehp.1003265)].

**Table 1:** Summary of existing studies on maternal–fetal transfer of total PFOA and total PFOS.

Mean cord:maternal serum concentration (correlation coefficient)		
Study	Sampling year	Location	Sample size	PFOA	PFOS
Needham et al. 2011		2000		Faroe Islands		12		0.72 (0.91)*a*		0.34 (0.82)*a*
Kim et al. 2011		2007		Korea		20		0.69 (0.88)*a*		0.36 (0.50)*b*
Fromme et al. 2010		2007–2009		Germany		27		0.70 (0.94)*b*		0.30 (0.89)*b*
Hanssen et al. 2010		2005–2006		South Africa		58		0.71 (0.67)*b*		0.45 (0.88)*b*
Monroy et al. 2008		2004–2005		Canada		101		0.81 (0.88)*a*		0.45 (0.83)*a*
Midasch et al. 2007		2003		Germany		11		1.26 (0.72)*b*		0.60 (0.42)*b*
Fei et al. 2007		1996–2002		Denmark		50*c*		0.55		0.29
						50*d*		0.68 (0.84)*a*		0.34 (0.72)*a*
Inoue et al. 2004		2003		Japan		15				0.32 (0.88)*a*
Present study		2007		Canada		20*d*		0.61 (0.63)*b*		0.33 (0.81)*b*
						20*e*		0.71 (0.76)*b*		0.36 (0.81)*b*
**a**Pearson correlation. **b**Spearman rank correlation. **c**Maternal serum was sampled in the first trimester. **d**Maternal serum was sampled in the second trimester. **e**TTE adjusted from 15 weeks to time of delivery (~ 40 weeks) using data from Monroy et al. (2008), whereby PFOS declined 10% and PFOA declined 12% between the 24th to 28th week and delivery.										

*Isomer-specific PFC analysis.* The isomer-specific HPLC-MS/MS method was adapted from Benskin et al. (2007). Briefly, 3 μL of the same extracts analyzed for total PFCs was injected onto a FluoroSep RP Octyl column (ES Industries, West Berlin, NJ, USA). Flow rate was 200 μL/min, and starting conditions were 60% A (water adjusted to pH 4.0 with ammonium formate) and 40% B (methanol). Initial conditions were held for 0.3 min, then ramped to 64% B by 1.9 min; increased to 66% B by 5.9 min, 70% B by 7.9 min, 78% B by 40 min, 88% B by 42 min and finally to 100% B by 45 min; and held until 60 min. Mass spectral data were collected using a 5000Q mass spectrometer (MDS Sciex, Concord, ON, Canada) equipped with an electrospray interface operating in negative-ion mode. Chromatograms were recorded by multiple reaction monitoring with 3–13 transitions per analyte.

*Quality control.* Triplicate recovery experiments were performed at two concentrations of native linear standards spiked to calf serum or dust [see Supplemental Material, Table 1 (http://dx.doi.org/10.1289/ehp.1003265)]. There are no mass-labeled internal standards for branched PFOS or PFOA isomers, so a standard addition experiment was done in dust to rule out possible matrix effects on the measured isomer profiles. Additionally, a vacuuming experiment was done to check whether off-gassing during vacuuming may bias the dust isomer profile. The results of these two experiments clearly showed that matrix effects and off-gassing during vacuuming were not a problem. The percent recovery during serum extraction was similar for all PFOS and PFOA isomers, such that the extraction step had no effect on the resulting isomer profiles (Benskin et al. 2007).

## Results and Discussion

*Total PFC concentrations in house dust.* All total PFCs, except for perfluorodecane sulfonate (PFDS), were log-normally distributed [Shapiro–Wilk test; for distributions, see Supplemental Material, Table 2 (http://dx.doi.org/10.1289/ehp.1003265)]. The three major PFCs in all dust samples (*n* = 18) were PFOA, PFOS, and PFHxA, with similar median values of 38, 37, and 35 ng/g, respectively. However, PFHxS exceeded PFOS in four samples. This pattern, whereby PFOA, PFOS, and PFHxA were the dominant PFCs, is similar to results from Strynar and Lindstrom (2008), who monitored U.S. house dust collected in 2001/2002 and found median PFOA, PFOS, and PFHxA concentrations of 142, 201, and 54.2 ng/g, respectively. The higher concentrations of PFOS and PFOA observed by Strynar and Lindstrom (2008) are understandable given that these dust samples were collected years earlier than in the present study, around the time of the phase-out of ECF C8 perfluorocarbon chemistries, although sampling strategy and geography may also have contributed to differences.

**Table 2:** TTE calculated from cord:maternal serum concentrations.

Compound	Arithmetic mean	Median	SD	Minimum	Maximum	*na*
Total, linear, and branched PFOS												
Total PFOS		0.33		0.31		0.09		0.20		0.53		20
*n*-PFOS		0.33		0.30		0.12		0.10		0.58		20
*Iso*- PFOS		0.36		0.34		0.14		0.09		0.60		20
5*m*-PFOS		0.53		0.52		0.18		0.25		0.93		20
5*m*-PFOS		0.53		0.52		0.18		0.25		0.93		20
4*m*-PFOS		0.55		0.52		0.19		0.20		0.85		20
3*m*-PFOS		0.67		0.68		0.23		0.21		1.12		20
1*m*-PFOS		0.87		0.88		0.23		0.36		1.24		20
Σ*m*_2_-PFOS		0.84		0.78		0.37		0.22		1.72		20
Total, linear, and branched PFOA												
Total PFOA		0.61		0.63		0.17		0.32		0.96		20
*n*-PFOA		0.62		0.61		0.20		0.26		1.00		20
*Iso*-PFOA		0.84		0.67		0.58		0.16		2.56		20
5*m*- PFOA		0.86		0.54		0.99		0.09		2.26		4
4*m*- PFOA		0.64		0.68		0.34		0.09		1.29		19
3*m*-PFOA		0.76		0.68		0.59		0.07		2.74		18
*tb*-PFOA		0.25		0.25		0.32		0.02		0.48		2
Other PFCs												
Total PFNA		0.41		0.38		0.17		0.13		0.78		20
Total PFDA		0.34		0.23		0.25		0.00		1.10		16
Total PFHxS		0.41		0.38		0.12		0.29		0.56		8
Values < 1 indicate higher concentrations in maternal serum; > 1.0, higher concentrations in cord serum. **a**Number of maternal–cord pairs that were available for calculating TTE. When concentrations were nondetect in maternal or cord samples, that pair was excluded in the analysis. Mean TTEs were always < 1.0, indicating lower concentrations in the cord serum than maternal serum (all *p* < 0.01). Linear PFOS and PFOA are denoted as *n*-PFOS and *n*-PFOA respectively.												

*PFC isomer profiles in house dust.* For PFOS, we detected six major branched isomers in dust: 1*m*-, 3*m*-, 4*m*-, 5*m*-, *iso*-, and Σ*m*_2_-PFOS. All the dust samples had PFOS isomer profiles that were very similar to the 3M Company ECF standard of PFOS, with a mean (± SD) branched isomer content of 30 ± 2.7%, and relatively low variability among individual branched isomers in various samples [Supplemental Material, Figure 3A, Table 3 (http://dx.doi.org/10.1289/ehp.1003265)]. This was not surprising given that the 3M Company produced the bulk of PFOS (Paul et al. 2009) and that the historical batch-to-batch variation of branched isomer content was small: 30 ± 0.8% branched PFOS in 18 lots over 20 years (Reagen WKL, Jacoby CB, Purcell RG, Kestner TA, Payfer RM, et al., unpublished data).

**Figure 3:**
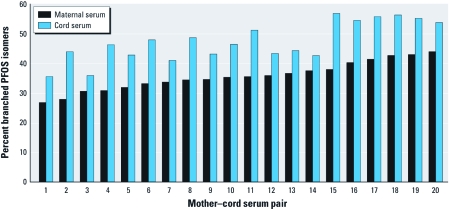
Percent branched PFOS isomers [Σbranched/(Σbranched + linear)] in 20 matched samples of maternal serum at 15 weeks of gestation and cord serum at delivery. Samples are arranged, from left to right, by increasing branched PFOS isomer content of the maternal sample.

Unlike PFOS, PFOA isomer profiles in dust were often substantially different from the 3M ECF PFOA standard. Although the relative profile among individual branched PFOA isomers was consistent among dust samples [Supplemental Material, Figure 3B, Table 3 (http://dx.doi.org/10.1289/ehp.1003265)], we observed an excess signal of linear PFOA in many of the samples compared with the 3M Company ECF standard. Like 3M Company PFOS, batches of 3M Company PFOA also had a consistent isomer composition: 22 ± 1.2% branched isomers in 18 lots over 20 years (Reagen WKL, Jacoby CB, Purcell RG, Kestner TA, Payfer RM, et al., unpublished data). Therefore, these observations suggest that a significant proportion of PFOA in these house dust samples came from a manufacturing source that used telomerization instead of ECF. We calculated the “percent telomer” PFOA in each dust sample from the excess signal of linear isomer in samples (*m*/*z* 413/369 transition) compared with 3M Company ECF PFOA. The percent telomer ranged from 0 to 95%, with a median of 31%, among all samples (Figure 1). The presence of telomer PFOA in the human household environment may partly explain why total PFOA in serum has declined so slowly after the phase-out of ECF perfluorooctyl chemistries by 3M Company in the United States [see Supplemental Material, Figure 1 (http://dx.doi.org/10.1289/ehp.1003265)] (Olsen et al. 2008). However, telomer PFOA also may be present in food, and telomer PFOA precursors used in food packaging may be absorbed and biotransformed to PFOA after ingestion (D’eon and Mabury 2011).

Isomer-specific chromatograms of house dust clearly indicate that other perfluorocarboxylates (i.e., PFCs with –CO_2_^–^ as a functional group) had only minor branched isomer content (data not shown). Authentic standards were not available for confirmation, so we identified peaks as branched isomers only when two characteristic MS/MS transitions responded at the same retention time. Most perfluorocarboxylates other than PFOA appeared exclusively linear, but in a minority of dust samples PFNA had up to four minor branched isomers, whereas PFHxA, PFDA, PFUnA, and PFDoA each had up to two minor branched isomers. The manufacturing sources of these particular branched perfluorocarboxylates cannot be confirmed because of limited information on their manufacturing sources and a lack of reference materials, but they may be residuals from ECF manufacturing of PFOS and PFOA. Perfluorosulfonates, such as PFDS, perfluoroheptane sulfonate, and PFHxS, are generally assumed to have been produced exclusively by ECF, and these all had major branched isomer content based on peak areas (data not shown). However, because reference materials were not available, we could not examine how closely they resembled authentic ECF manufacturing sources.

*Total PFCs in maternal and cord sera.* Concentrations of total PFOS (*n* = 20), PFOA (*n* = 20), PFNA (*n* = 20), PFDA (*n* = 16), and PFHxS (*n* = 8) in maternal serum were always significantly higher (*p* < 0.01) than in cord serum, consistent with previous maternal–fetal transfer studies of PFOS and PFOA (Table 1). The major total PFCs in maternal and cord sera were PFOS, PFOA, PFHxS, and PFNA, similar to previous findings (Inoue et al. 2004; Midasch et al. 2007; Monroy et al. 2008). In the present study, the mean concentrations of PFOS, PFOA, PFHxS, PFNA, and PFDA in maternal serum (cord serum) were 5.5 (1.8), 1.8 (1.1), 1.7 (0.7), 0.9 (0.4), and 0.4 (0.1) ng/mL, respectively. We detected PFUnA, PFDoA, and perfluorotetradecanoate (PFTA) (detection limit, 0.1 ng/mL) in six, two, and three maternal samples, respectively, but in no cord samples.

We estimated transplacental transfer efficiencies (TTEs) by dividing the PFC concentrations in cord serum at delivery by maternal serum concentration at 15 weeks of gestation for each mother–cord pair (Table 2). Mean TTEs were always < 1.0, indicating lower concentrations in cord serum than in maternal serum (all *p* < 0.01). Overall, the PFOS and PFOA TTEs were within the range reported in the literature. However, it is likely that our TTEs slightly underestimate actual TTE values because they do not reflect hematologic changes that occur later in pregnancy, including expansion of total plasma volume (Whittaker et al. 1996). Such an effect was shown by Monroy et al. (2008), who reported lower serum PFOS and PFOA levels in maternal serum samples collected at delivery versus 24th through 28th weeks of gestation, and by Fei et al. (2007), who reported that cord:maternal ratios based on maternal serum samples were higher when collected during the second trimester than during the first trimester (Table 1). We used the data from Monroy et al. (2008) (see Table 1 notes) to estimate time-of-delivery maternal serum concentrations, based on our 15-week data, but this had little effect on the resulting TTEs, and both adjusted and unadjusted TTE values were within the range of TTEs reported previously (Table 1).

A comparison of TTEs among the three major perfluoroalkyl carboxylates (PFOA, PFNA, and PFDA) suggests that the longer-chain carboxylates were more efficiently blocked by the placental barrier (Figure 2A), consistent with the results of Kim et al. (2011). The same trend was also evident for the two major perfluorosulfonates (PFHxS and PFOS; Figure 2B). Overall, shorter-chain PFCs crossed the placenta more efficiently than did longer-chain PFCs, consistent with the findings of Needham et al. (2011).

*PFC isomer profiles in maternal and cord sera.* The percent branched content of total PFOS was consistently and significantly higher in cord serum than in corresponding maternal serum and dust samples (Figure 3). Branched PFOS isomers contributed 27–44% (median, 36%) of total PFOS in maternal serum and 36–54% (median, 46%) in cord serum. A paired *t*-test indicated statistically greater proportions of branched PFOS in the cord serum (*p* < 0.01). Overall, all branched PFOS isomers were transferred more efficiently (median TTEs of the different branched isomers, 0.34–0.88) than was the linear isomer (median TTE, 0.30) (Table 2). This is similar to results of the Hanssen et al. (2010) study, which found a statistically greater relative abundance of linear PFOS in maternal serum than in cord serum relative to total branched PFOS isomers (*p* < 0.05 by Wilcoxon’s signed rank test).

Unlike Hanssen et al. (2010), who quantified total branched PFOS isomers together, we analyzed individual branched isomers, and results suggest a structure–activity relationship for TTE. Specifically, among the perfluoromethyl PFOS branched isomers, TTE increased as the branching point moved closer to the sulfonate moiety: 1*m* > 3*m* > 4*m* ≈ 5*m* > *iso* (Figure 2C). In fact, for 1*m*-, 3*m*-, and particularly Σ*m*_2_-PFOS, the concentrations were sometimes higher in cord serum than in corresponding maternal serum (resulting in maximum TTE values > 1.0), which was never the case for total PFOS or linear PFOS (Table 2).

Branched PFOA isomers contributed 0.43–4.3% (mean, 1.9%) of total PFOA in maternal serum and 0.71–5.7% (mean, 2.2%) in cord serum. Such highly linear isomer profiles of PFOA in human serum have previously been reported (De Silva and Mabury 2006), yet it is important to note that these cannot be used to quantitatively assess exposure sources (i.e., telomer vs. electrochemical) because in animal models the branched isomers of PFOA are accumulated to a lesser extent than are linear PFOA (De Silva et al. 2009). No structure–activity relationship was evident for PFOA isomers (Figure 2D), but a paired *t*-test indicated significantly higher total branched PFOA isomers in cord serum than in maternal serum (*p* = 0.02). In some cases, the concentrations of 5*m*-, 4*m*-, and 3*m*-PFOA were higher in the cord serum than in corresponding maternal serum (resulting in maximum TTE > 1.0), which was never the case for total PFOA or linear PFOA (Table 2).

Passive diffusion is often the mechanism by which chemicals cross the placental barrier (Syme et al. 2004), so the TTE of hydrophilic compounds is generally lower than for hydrophobic compounds (Van der Aa et al. 1998). Based on earlier elution in reverse-phase chromatography, branched PFOS isomers are anticipated to be more hydrophilic than linear PFOS, and short-chain carboxylates (e.g., PFOA) should be more hydrophilic than longer-chain carboxylates (e.g., PFNA and PFDA), so the present results are unexpected. However, perfluorinated acids are highly protein bound in serum (Jones et al. 2003), and the dynamics of protein binding are likely to influence TTE. For example, if the binding affinity of linear PFOS to maternal serum protein is higher than for branched PFOS isomers, a higher free fraction of branched PFOS would be available to cross the placenta.

For the major PFCs in maternal serum, we examined whether the branched isomer content was correlated to the branched isomer content in the corresponding house dust sample. Although we did not observe a significant correlation between serum and dust branched isomer content for PFOS (Spearman correlation coefficient = –0.10, *p* = 0.35) or PFHxS (Spearman correlation coefficient = –0.11, *p* = 0.33), we found a borderline significant correlation for PFOA (Spearman correlation coefficient = 0.35, *p* = 0.08). However, we cannot confirm that dust was a source of branched PFOA isomers in these women, given the small sample size (*n* = 20) and the potential contribution of other sources of exposure, including diet, water, and air (Haug et al. 2011).

In contrast with expectations, we observed a higher mean percentage of branched PFOS isomers in maternal serum [36%; 95% confidence interval (CI): 33.6, 38.2%] than in historic 3M Company ECF PFOS (30%; 95% CI: 29.3, 30.7%) (Reagen WKL, Jacoby CB, Purcell RG, Kestner TA, Payfer RM, et al., unpublished data) or in house dust samples (30%; 95% CI: 28.6, 31.3%). A paired *t*-test showed significantly higher branched PFOS content in maternal serum than in house dust (6% higher; 95% CI: 3.1, 8.9%; *p* < 0.001). Studies in rodents show that branched PFOS isomers are no more bioaccumulative than linear PFOS (De Silva et al. 2009), so it would seem pharmacokinetically impossible to accumulate > 30% branched PFOS isomers if the only source of exposure was ECF PFOS. Nonetheless, Karrman et al. (2007) and Hanssen et al. (2010) also reported high proportions of branched PFOS isomers in human serum. Although it is possible that PFOS isomer pharmacokinetics in humans are opposite that in rats (De Silva et al. 2009) or that some humans are exposed to an unusually high branched PFOS source in the diet, an alternative explanation is that a significant proportion of the PFOS body burden comes from metabolism of PFOS precursors. Benskin et al. (2009) demonstrated that branched isomers of a PFOS precursor could be biotransformed at greater rates than the corresponding linear precursor, and Haug et al. (2011) found a significant association between PFOS precursors in air and increasing branched PFOS content of serum. In the present samples, maternal and cord serum PFOS concentrations were higher (*p* < 0.01 for maternal serum, *p* = 0.01 for cord serum) when we detected *N*-methyl perfluorooctanesulfonamidoacetate (a PFOS precursor) in the same sample, but we found no significant association between total dust PFOS precursors and the branched PFOS content of serum (*p* = 0.47).

*Study limitations.* One limitation of this study is the relatively small sample size. Larger studies are recommended to elucidate the relative importance of ECF- and telomer-derived sources of PFCs to humans in other areas. The present study was not designed to test whether PFC signatures in dust were responsible for PFC signatures in maternal or cord serum; rather, it was an exploratory investigation of the variability of isomer profiles in dust to elucidate manufacturing sources, and of the variation of isomer profiles between maternal and cord samples to examine whether branched isomers crossed the placenta to different extents.

A second limitation was that the time of sampling of pregnant women (15 weeks) was relatively early in the pregnancy, and it is not clear whether the isomer profile might have been different at time of delivery. For two women in our study we also analyzed 18-week serum samples, and total PFOS and individual PFOS isomers were not substantially different over these 3 weeks (data not shown). Although this is a narrow window of time, it is not an insignificant period because hematologic indices change significantly beginning as early as the 7th week of pregnancy, including expansion of total blood plasma volume by 16% between 12 and 20 weeks (Whittaker et al. 1996).

## Conclusion

Both ECF and telomer manufacturing sources contributed to household dust PFOA concentrations in this exploratory study. Some homes with the highest PFOA dust concentrations had a near exclusive telomer PFOA signal, and such results may help explain why PFOA continues to be a major contaminant of human serum despite the ECF PFOA phase-out. Larger-scale studies that examine manufacturing sources while simultaneously accounting for dietary pathways would be beneficial. It is recognized that such investigations are technically challenging because isomer profiles in biological samples (i.e., food) may bias source apportionment due to differential uptake of the various isomers. The TTE of PFCs was inversely related to chain length, and TTEs suggest that most branched PFOA and PFOS isomers crossed the placenta to a greater extent than the corresponding linear isomer. In some cases, minor PFOA and PFOS branched isomers were more concentrated in cord serum than maternal serum, indicating that isomer-specific analysis should be performed in future studies of PFCs and birth outcomes.

## Supplemental Material

(224 KB) PDF
