# Supplementary material for: Isomer Profiles of Perfluorochemicals in Matched Maternal, Cord, and House Dust Samples: Manufacturing Sources and Transplacental Transfer
Source: Environ Health Perspect. 2011 Jul 14;119(11):1659–64. doi: 10.1289/ehp.1003265 (PMC3226492; doi:10.1289/ehp.1003265)
Supplement: (224 KB) PDF [file ehp.1003265.s001.pdf]

SUPPLEMENTAL MATERIAL

Isomer Profiles of Perfluorochemicals in Matched Maternal, Cord and House Dust Samples: Manufacturing Sources and Transplacental Transfer

Sanjay Beesoon,[1] Glenys M. Webster,[2] Mahiba Shoeib,[3] Tom Harner,[3] Jonathan P. Benskin,[1] and Jonathan W. Martin[1]

[1]Division of Analytical & Environmental Toxicology, Department of Laboratory Medicine & Pathology, University of Alberta, Edmonton, AB, Canada; [2]Centre for Health & Environment Research, School of Environmental Health, University of British Columbia, Vancouver, Canada; [3]Science and Technology Branch, Environment Canada, Toronto, Ontario, Canada.

TABLE OF CONTENTS

	Page
Figure 1 - Trend in serum PFOS and PFOA in the United States	2
Figure 2 - Structural formula of PFOS and PFOA linear and branched isomers	3
Figure 3 - Isomer profiles in a house dust sample for PFOS and PFOA	4
Table 1 - Spike and recovery of native PFCs in serum and dust samples	5
Table 2 - Descriptive statistics of total PFCs in dust samples	6
Table 3 - Descriptive statistics on individual isomers of PFOS and PFOA in dust samples	7

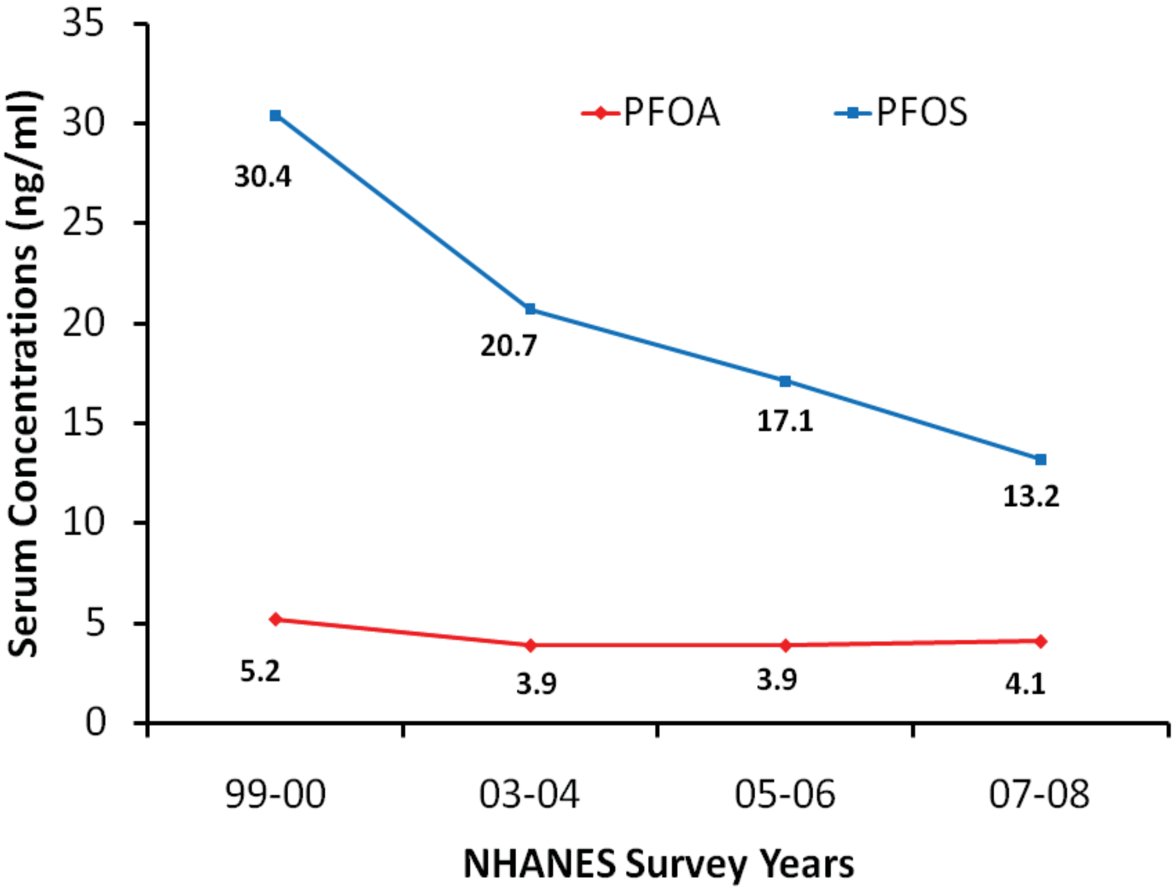

Supplemental Material, Figure 1. Trend in the geometric mean concentrations of PFOA and PFOS in the blood of Americans for the 10 year period from 1999 to 2008. PFOS has consistently declined, whereas PFOA has not declined since 2003/2004 and may be increasing. Data for the first 3 NHANES surveys are published data by the CDC, while the geometric means for 2007-2008 were calculated (after weight adjustment) using SAS data files available on the website of the National Center for Health Statistics of the CDC.

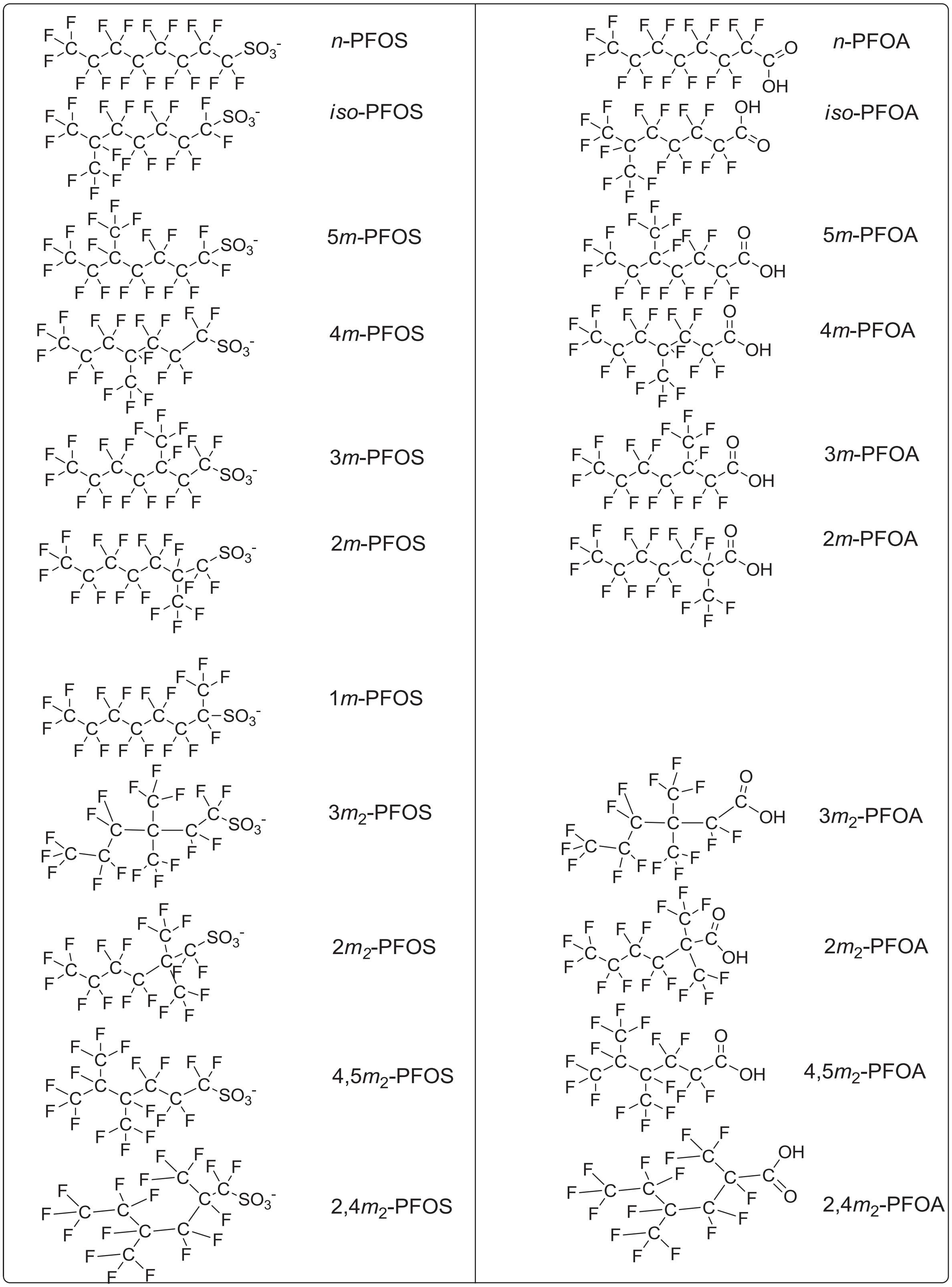

Supplemental Material, Figure 2. Structures of PFOS and PFOA linear and branched isomers. Note that 1*m*-PFOA does not exist. For geminal diperfluoromethyl-PFOS and geminal diperfluoromethyl-PFOA isomers, only the 3m_2 and 2m_2 are shown, although other such structures exist. Similarly for non-geminal diperfluoromethyl PFOS and PFOA only the 4,5 m_2 and 2,4 m_2 are shown, but other structures exist. In the isomer specific analysis, all the diperfluoromethyl PFOS isomers are grouped together and labeled as Σm_2-PFOS. Other nomenclature systems have been proposed (Rayne et al. 2008).

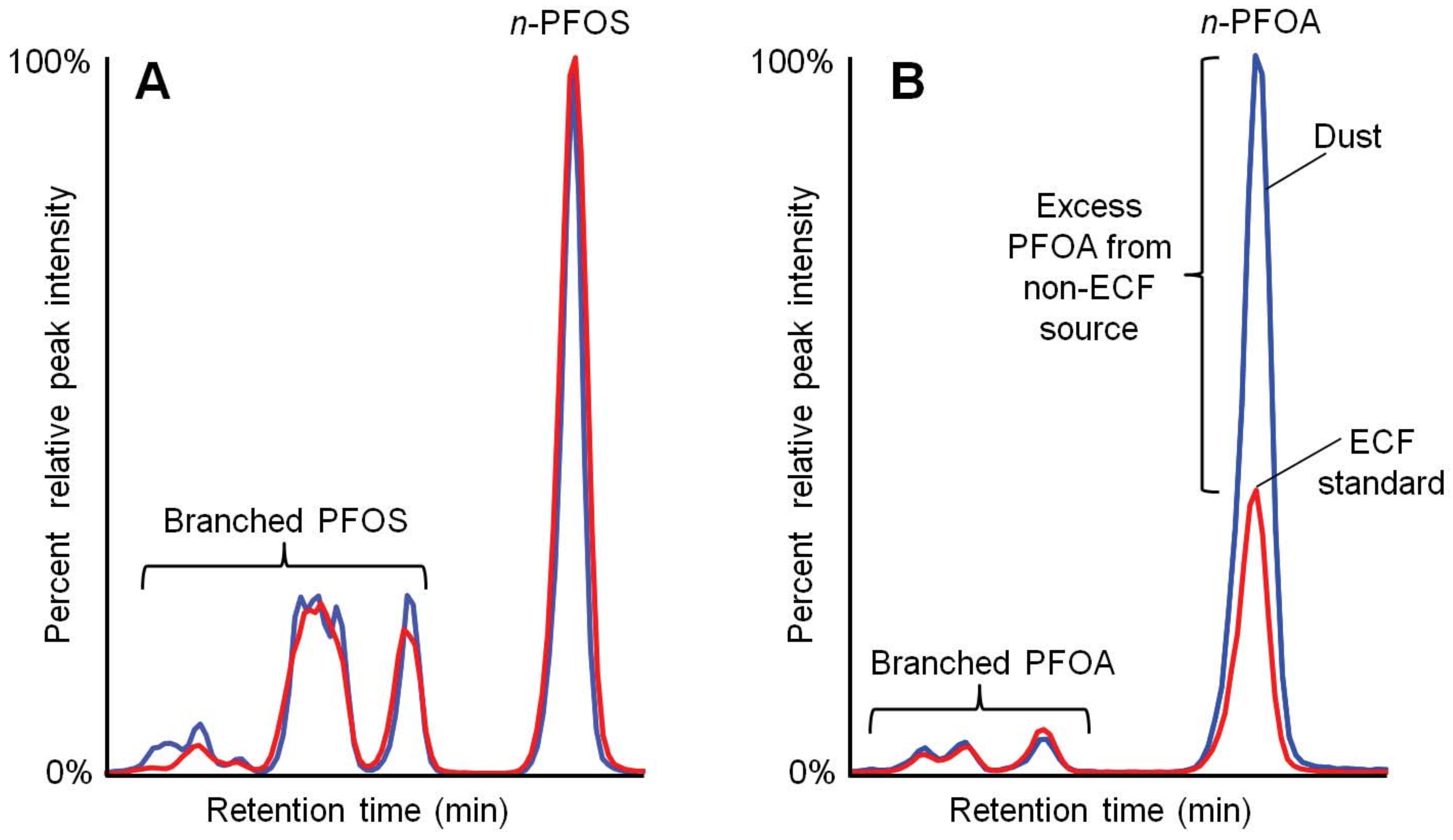

Supplemental Material Figure 3. Isomer profiles in a house dust sample for (A) PFOS (*m/z* 499/80) and (B) PFOA (*m/z* 413/369). Red traces represent the isomer profile in a 3M ECF standard, while the blue trace represents the isomer profile in a dust sample. Each profile was normalized to the response of the branched isomers, such that the relative amount of linear isomer in each case is easily compared.

Supplemental Material, Table 1. Spike and recovery of native PFCs in serum and dust. Experiments were done in triplicate in each case and figures presented here represent arithmetic means.

PFCs	Calf serum spiked at 0.5 ng/ml		Calf serum spiked at 10 ng/ml		Dust spiked at 60 ng/g	
	Mean % Recovery ±S.D	Range	Mean % Recovery ±S.D	Range	Mean % Recovery ± S.D	Range
PFBS	93.2 ± 16.9	79.4-128	-	-	71.7 ± 8.1	64.0 - 83.0
PFHxS	79.0 ± 13.0	61.8-94.8	91.8 ± 12.5	75 – 112	90.2 ± 12	72.1 - 105
PFHpS	95.5 ± 15.1	76.8-123	-	-	92.3 ± 27	46.8 - 116
PFOS	82.7 ± 16.8	52.0-99.3	93.1 ± 11.9	82 – 117	107 ± 14	89.9 - 121
PFDS	109 ± 8.7	93.3-120	64.0 ± 8.2	52 – 74	32.2 ± 15	14.7 - 48.9
PFBA	-	-	-	-	74.3 ± 8.9	60.1 - 84.7
PFPeA	-	-	-	-	83.7 ± 11	65.7 - 95.0
PFHxA	94.2 ± 9.3	78.3-108	-	-	128 ± 9	119 - 141
PFHpA	78.1 ± 10.7	66.2-97.4	-	-	97.9 ± 21	72.6 - 130
PFOA	82.5 ± 9.4	67.1-99.1	95.3 ± 15.5	68 – 129	83.0 ± 5.3	74.6 - 88.5
PFNA	94.4 ± 5.4	84.7-102	94.8 ± 8.3	74 – 101	93.1 ± 20	72.0 - 117
PFDA	81.9 ± 7.6	71.6-93.8	96.9 ± 4.6	91 – 106	102 ± 9	92.1 - 112
PFUnA	92.8 ± 7.6	81.6-106	93.8 ± 10.4	78 – 116	107 ± 11	95.1 - 118
PFDoA	102 ± 4.9	97.3-113	93.3 ± 5.8	83 – 101	95.6 ± 6.3	91.3 - 106
PFTrA	122 ± 6.9	115.0-133	54.0 ± 11.0	33 - 70	79.2 ± 17	59.9 - 106
PFTA	105 ± 7.4	97.2-117	-	-	42.1 ± 24	10.2 - 76.3
FOSA-M	108 ± 7.6	92.3-116	-	-	42.7 ± 4.9	38.2 - 50.9

Supplemental Material, Table 2. Summary statistics of total PFC concentrations (ng/g, non isomer specific) in house dust samples (n=18 unless otherwise noted). To compute the descriptive statistics, values less than limit of detection (LOD) have been replaced by LOD/2.

	Min	Max	Median	Mean	Geometric Mean	% above LOD
			Perfluoroalkyl sulfonates			
PFBS	<0.5	48	<0.5	6.1	0.7	28
PFHxS	2.9	1300	14	140	21	100
PFHpS	<0.5	46	<0.5	4.1	0.6	22
PFOS	<0.5	1300	37	180	39	94
PFDS	<0.5	5.1	2.1	2.2	1.8	94
			Perfluoroalkyl carboxylates			
PFBA	<0.5	42	2.6	9.2	3.6	94
PFPeA	<0.5	93	5.2	17	4.9	83
PFHxA	2.3	390	35	77	33	100
PFHpA	1.4	320	21	55	19	100
PFOA	4.3	820	38	120	50	100
PFNA	1.4	220	15	44	18	100
PFDA	1.7	250	15	44	16	100
PFUA	<0.5	240	6.1	31	8.0	94
PFDoA	1.4	160	10	36	13	100
PFTrA	<0.5	67	2.4	9.9	2.3	78
PFTA	<0.5	24	3.3	6.5	3.3	94
			Perfluoroalkyl sulfonamides			
PFOSA	<0.5	<0.5	<0.5	<0.5	0.3	0
NMeFOSA (n=16)	1.2	13.8	2.3	3.0	2.5	100
NEtFOSA (n=16)	<0.06	2.8	0.15	0.55	0.14	50
NMeFOSAA	<0.5	440	1.2	36	2.3	50
NEtFOSAA	3.2	240	27	58	32	100
NMeFOSE (n=16)	15	910	49	152	65	100
NEtFOSE (n=16)	<0.02	190	10	14	5.3	88

Supplementary Material, Table 3. Descriptive statistics of the percentage of individual isomers of PFOS and PFOA in house dust samples (N=18), and mean (n=3 injections) of the 3M ECF standard.

	**PFOS**							**PFOA**						
	Linear	*iso*	5*m*	4*m*	3*m*	1*m*	$\sum m_2$	Linear	*iso*	5*m*	4*m*	3*m*	$\sum m_2$	*tb*
Mean	69.4	17.6	4.55	0.61	5.08	0.81	2.13	82.0	5.54	4.77	3.12	3.21	0.80	0.56
SD[a]	4.81	3.60	1.01	0.16	1.78	0.77	0.68	11.2	3.28	3.57	3.01	1.77	0.59	0.4
Median	70.7	16.2	4.33	0.58	5.1	0.58	2.00	84.2	4.99	4.21	2.69	3.37	0.68	0.55
Min	53.4	14.8	3.20	0.42	<LOD[b]	<LOD	1.34	59.0	0.46	<LOD	<LOD	0.19	0.05	0.03
Max	73.5	27.6	7.73	1.14	9.65	2.26	4.31	98.6	12.2	11.6	8.15	6.17	2.38	1.62
Mean ECF Std	70.2	18.1	4.83	0.58	4.18	0.46	1.69	73.4	9.05	7.35	5.80	2.98	0.74	0.67

[a] The low relative standard deviations (SDs) associated with the mean percentage of PFOS isomers in the 18 dust samples suggest a similar ECF source, contrasted to the high SD for linear PFOA, suggesting a mixture of ECF and telomer sources.
[b] LOD-Limit of detection.
